# Melatonin Attenuates ox-LDL-Induced Endothelial Dysfunction by Reducing ER Stress and Inhibiting JNK/Mff Signaling

**DOI:** 10.1155/2021/5589612

**Published:** 2021-03-04

**Authors:** Peng Li, Changlian Xie, Jiankai Zhong, Zhongzhou Guo, Kai Guo, Qiuyun Tu

**Affiliations:** ^1^Department of Gerontology, The Fifth Affiliated Hospital, Sun Yat-sen University, Zhuhai, Guangdong Province 519000, China; ^2^Guangdong Provincial Key Laboratory of Biomedical Imaging, The Fifth Affiliated Hospital, Sun Yat-sen University, Zhuhai, Guangdong Province 519000, China; ^3^Intensive Care Unit, The Traditional Chinese Medicine Hospital, Zhongshan, Guangdong Province 528400, China; ^4^Department of Cardiology, Shunde Hospital, Southern Medical University (The First People's Hospital of Shunde), Foshan, Guangdong Province 528308, China; ^5^Department of Cardiology, Nanfang Hospital, Southern Medical University, Guangzhou, Guangdong Province 510515, China; ^6^Cardiovascular Medicine Department, Guangdong Second Provincial General Hospital, Guangzhou, Guangdong Province 510317, China

## Abstract

Endothelial dysfunction, which is characterized by damage to the endoplasmic reticulum (ER) and mitochondria, is involved in a variety of cardiovascular disorders. Here, we explored whether mitochondrial damage and ER stress are associated with endothelial dysfunction. We also examined whether and how melatonin protects against oxidized low-density lipoprotein- (ox-LDL-) induced damage in endothelial cells. We found that CHOP, GRP78, and PERK expressions, which are indicative of ER stress, increased significantly in response to ox-LDL treatment. ox-LDL also induced mitochondrial dysfunction as evidenced by decreased mitochondrial membrane potential, increased mitochondrial ROS levels, and downregulation of mitochondrial protective factors. In addition, ox-LDL inhibited antioxidative processes, as evidenced by decreased antioxidative enzyme activity and reduced Nrf2/HO-1 expression. Melatonin clearly reduced ER stress and promoted mitochondrial function and antioxidative processes in the presence of ox-LDL. Molecular investigation revealed that ox-LDL activated the JNK/Mff signaling pathway, and melatonin blocked this effect. These results demonstrate that ox-LDL induces ER stress and mitochondrial dysfunction and activates the JNK/Mff signaling pathway, thereby contributing to endothelial dysfunction. Moreover, melatonin inhibited JNK/Mff signaling and sustained ER homeostasis and mitochondrial function, thereby protecting endothelial cells against ox-LDL-induced damage.

## 1. Introduction

Endothelial dysfunction is associated with a variety of cardiovascular disorders such as ischemic heart disease, myocardial infarction, postinfarction heart remolding, diabetic cardiomyopathy, and hypertension [1–3]. The cardioprotective effects of several clinical drugs, including statins, aspirin, and clopidogrel, are reportedly associated with endothelial protection [4, 5]. In addition, many risk factors, such as oxidized low-density lipoprotein (ox-LDL), blood flow shear force, inflammation cytokines, oxidative stress, and septic shock, contribute to endothelial dysfunction. Among these risk factors, endothelial cells are particularly vulnerable to ox-LDL-induced stress through unknown mechanisms [6–8]. Increases in ox-LDL result in decreased proliferative ability, impaired migratory response, increased apoptotic index, and reduced regenerative capability in endothelial cells [9–11]. Although many studies have examined the relationship between ox-LDL and pathological alterations in endothelial cells *in vivo* and *in vitro*, the key molecular mechanisms underlying ox-LDL-associated endothelial dysfunction have not been fully explained, and few effective therapeutic drugs are available for patients with endothelial dysfunction.

Endoplasm reticulum (ER) stress is an adaptive response that regulates protein synthesis and folding within cells [6]. Moderate activation of ER stress is associated with timely removal of damaged or unfolded proteins, thereby contributing to protein quality control [12]. Interestingly, excessive induction of ER stress contributes to abnormal degradation of damaged proteins and activation of caspase-dependent cell apoptosis [13]. ER-related cell apoptosis is characterized by increased levels of CHOP and caspase-12 [14, 15]. In addition, previous studies have reported a close relationship between endothelial dysfunction and ER stress [16]. Excessive ER stress promotes calcium overload, leading to spasms in endothelial cells [17]. Migratory response and angiogenesis might also be affected by ER stress because of its connections to synthesis and secretion of proteins like vascular endothelial growth factor (VEGF), which is necessary for angiogenesis [18, 19]. In addition to the ER, the mitochondria are also responsible for energy supply and redox balance within endothelial cells [20, 21]. Impaired mitochondrial function resulting from mitochondrial fragmentation and decreased mitochondrial autophagy can act as an upstream regulator of endothelial dysfunction [22, 23]. Many physiological processes, such as endothelial cell movement, growth, proliferation, and regeneration, are highly dependent on mitochondrial function [24–26]. Mitochondrial damage reduces the available ATP in endothelial cells and therefore contributes to endothelial dysfunction. Recent studies report that mitochondrial damage is regulated by the JNK/Mff signaling pathway in the context of cardiac ischemia-reperfusion injury [27–29]. However, this mechanism has not been verified in ox-LDL-treated endothelial cells.

Melatonin is a classical cardioprotective drug with multiple effects on endothelial function. Melatonin treatment attenuates microvascular damage during ischemia-reperfusion injury [30, 31]. Melatonin also attenuates endothelial dysfunction in diabetic cardiomyopathy [32]. Furthermore, melatonin inhibits oxidative stress, calcium overload, and inflammation response in endothelial cells under different disease models [33, 34]. In the present study, we examined whether ox-LDL-induced endothelial dysfunction is associated with ER stress and JNK/Mff signaling pathway activation. We also conducted experiments to understand whether melatonin improves endothelial function by altering ER stress and the JNK/Mff signaling pathway.

## 2. Materials and Methods

### 2.1. Cell Line Culture

Human umbilical vein endothelial cells (HUVECs) were purchased from American Type Culture Collection (ATCC; Manassas, VA, USA). Cells were cultured in RPMI 1640 (HyClone, Logan, UT, USA) containing 10% FBS (Gibco, Rockville, MD, USA) and maintained in a 37°C, 5% CO_2_ incubator [35]. HUVECs were incubated with oxidized low-density lipoprotein (ox-LDL) at a concentration of 50 *μ*g/mL according to a recent report [36]. To observe the protective effects of melatonin on ox-LDL-treated HUVECs, melatonin was added to the culture medium at a concentration of 5 *μ*M based on a previous study [37].

### 2.2. 3-(4,5-Dimethyl-2-thiazolyl)-2,5-diphenyl-2-H-tetrazolium Bromide (MTT) Assay

HUVECs were plated on 96-well plates. After 24 h, 48 h, and 72 h of proliferation, 10 *μ*L MTT (Beyotime, Shanghai, China) was added to each well for 4 h. The cells were then treated with 100 *μ*L dimethyl sulfoxide (DMSO; Sigma) for 2 h. Finally, the optical density (OD) value at 490 nm was measured using a spectrophotometer [38].

### 2.3. CCK-8 Assay

HUVECs were seeded onto 96-well plates and then incubated for 24, 48, 72, or 96 h. the CCK-8 reagent was then added to each well and then incubated for 4 h. The absorbance value of each well was measured at 490 nm [39].

### 2.4. Enzyme-Linked Immunosorbent Assay (ELISA)

HUVECs at a density of 1 × 10^5^ cells/well were cultured in 96-well plates at 37°C with 5% CO_2_ for 24 hours. Cell culture supernatant was then collected, and GSH, GPX, and SOD levels were quantified using the corresponding ELISA kits purchased from R&D (San Diego, CA, USA) [40]. OD values at 450 nm absorbance were measured using an ELX808 Absorbance Reader (BioTek, London, UK) [41].

### 2.5. Mitochondrial Membrane Potential (MMP) Assessment

MMP was assessed using the JC-1 mitochondrial membrane potential assay kit (C2006, Beyotime, Shanghai, China) as described in a previous study [42]. HUVECs were collected after treatment with melatonin in the presence of ox-LDL. The cells were then incubated with 5 *μ*M JC-1 staining kit reagent at 37°C for 30 minutes in the dark [43]. Finally, cells were analyzed using the Guava easyCyte Benchtop Flow Cytometer (BR168323; Luminex, Austin, TX, USA) with Kaluza C Analysis Software (version 2.1, Beckman Coulter, Indianapolis, IN, USA) [44].

### 2.6. Western Blot

Protein expression levels were determined via western blot as previously described [45]. After cell collection, protein lysis and extraction were performed using RIPA lysis buffer (R0278, Sigma-Aldrich, USA), and the concentration of extracted protein was measured using the Bicinchoninic acid (BCA) protein assay kit (AR0146, Boster Bio, Pleasanton, CA, USA) [46]. Then, 20 *μ*g protein lysates samples were subjected to sodium dodecyl sulfate-polyacrylamide gel electrophoresis (SDS-PAGE; P0012AC, Beyotime, China) and transferred onto polyvinylidene fluoride (PVDF) membranes (FFP36, Beyotime, China), which were blocked with fat-free milk (5%) for 2 hours and incubated with primary antibodies at 4°C overnight; *β*-actin was used as internal control [47, 48]. The membranes were then incubated in secondary horseradish peroxidase- (HRP-) conjugated antibodies at room temperature for 1 hour and washed three times using tris-buffered saline Tween (TBST, T196393, Aladdin, China). Protein bands were visualized using the enhanced chemiluminescence (ECL) kit (P0018FS, Beyotime, China). Gray band density values were analyzed in the iBright CL1500 Imaging System (A44240, Thermo Fisher Scientific, USA) and calculated using ImageJ (version 5.0, Bio-Rad, Hercules, CA, USA) [49].

### 2.7. Detection of Caspase-3 Activity

Caspase-3 activity was evaluated using the caspase-3 activity assay kit (Beyotime) as previously described [50]. Briefly, HUVECs were seeded on 24-well plates and incubated with melatonin in the presence of ox-LDL for 48 h. The cells were then collected, and caspase-3 activity assay kit was measured [51].

### 2.8. Transwell Assay

For the migration assay, HUVECs were placed in the upper chamber of transwells [52]. For the invasion assay, the upper chamber was treated with Matrigel before cells were added. After incubation for 48 h, cells that had migrated to or invaded the lower chamber were stained with 0.1% crystal violet and quantified using a microscope [53].

### 2.9. Quantitative Real-Time Polymerase Chain Reaction (qRT-PCR)

Samples were incubated with TRIzol reagent (Invitrogen) to extract total RNA [54]. CRNDE, miR-4262, and ZEB1 cDNA were synthesized using the All-in-One™ cDNA Synthesis Kit (FulenGen, Guangzhou, China) [55]. Subsequently, qRT-PCR was performed using SYBR green (Applied Biosystems, Foster City, CA, USA). Glyceraldehyde-3-phosphate dehydrogenase (GAPDH) was used as an internal reference (56).

### 2.10. Statistical Analysis

All experiments were repeated three times. Data are presented as means ± standard deviation (SD). SPSS 17.0 software was used for statistical analyses. Student's *t*-tests and one-way analyses of variance (ANOVA) were used to identify differences between groups. *p* < 0.05 was considered statistically significant.

## 3. Results

### 3.1. Melatonin Attenuates ox-LDL-Induced Endothelial Dysfunction

**Figure 1:**
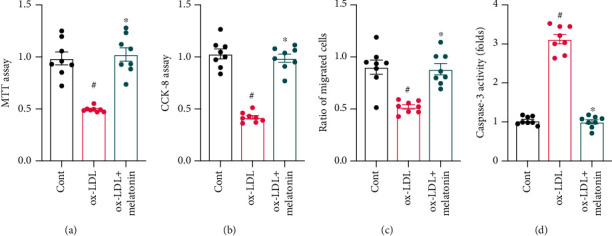
Melatonin attenuates ox-LDL-induced endothelial dysfunction. (a) HUVECs were treated with melatonin in the presence of ox-LDL. Cell viability was measured via MTT assay. (b) CCK-8 was used to measure the proliferative capacity of HUVECs after ox-LDL treatment. (c) A transwell assay was used to evaluate cell migratory response. (d) ELISA was used to analyze caspase-3 activity. ^#^*p* < 0.05 vs. the control group, ^∗^*p* < 0.05 vs. the ox-LDL group.

After ox-LDL was administered to induce endothelial dysfunction, melatonin was added to the growth medium to evaluate any protective actions. First, cell viability was measured using an MTT assay. As shown in Figure 1(a), compared to the control group, endothelial cell viability was significantly reduced by exposure to ox-LDL. Interestingly, melatonin reversed this ox-LDL-induced cell damage. Endothelial cell proliferation capacity was also measured in a CCK-8 assay. As shown in Figure 1(b), compared to the control group, ox-LDL exposure progressively reduced endothelial cell proliferation. Again, melatonin reversed this ox-LDL-induced effect by increasing endothelial cell proliferative capacity. Migratory response in endothelial cells is also important for angiogenesis. A transwell assay was therefore used to analyze alterations in endothelial cell migratory response. As shown in Figure 1(c), compared to the control group, ox-LDL significantly reduced the number of endothelial cells that migrated, and melatonin reversed this effect. Finally, cell apoptosis was monitored using a caspase-3 activity assay. As shown in Figure 1(d), compared to the control group, caspase-3 activity in endothelial cells was significantly reduced in the ox-LDL group, suggesting that ox-LDL treatment induced cell apoptosis. Finally, melatonin treatment prevented ox-LDL-induced upregulation of caspase-3 activity, suggesting that melatonin has antiapoptotic effects in the presence of ox-LDL.

### 3.2. Melatonin Alleviates ox-LDL-Induced ER Stress

**Figure 2:**
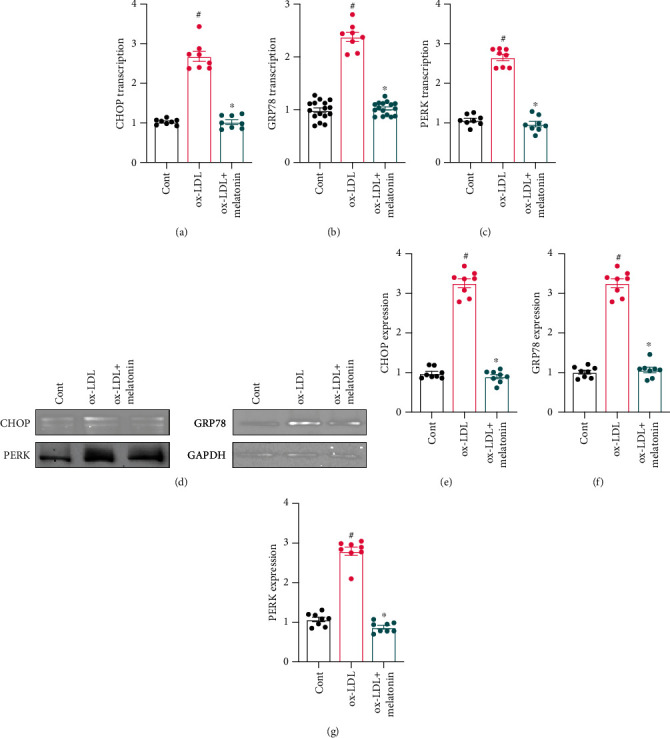
Melatonin alleviates ER stress during ox-LDL treatment. (a–c) HUVECs were treated with melatonin in the presence of ox-LDL. qPCR was then used to measure CHOP, GRP78, and PERK transcription. (d–g) Proteins were isolated from treated HUVECs, and expression of CHOP, GRP78, and PERK proteins was evaluated using western blots. ^#^*p* < 0.05 vs. the control group, ^∗^*p* < 0.05 vs. the ox-LDL group.

Changes in ER stress were evaluated to identify the molecular mechanism underlying ox-LDL-mediated endothelial dysfunction. First, ER stress biomarkers were analyzed by qPCR. As shown in Figures 2(a)–2(d), compared to the control group, transcription of CHOP, GRP78, and PERK was significantly elevated after ox-LDL treatment. However, melatonin inhibited this upregulation. To confirm that transcriptional activation of ER stress biomarkers is also associated with upregulation of ER stress-related proteins, western blots were used to quantify expression of proteins involved in ER stress initiation, augmentation, and execution. As shown in Figures 2(e)–2(g), compared to the control group, CHOP, GRP78, and PERK protein expressions rapidly increased as a result of ox-LDL treatment. Furthermore, melatonin treatment inhibited upregulation of ER stress-related proteins, suggesting that melatonin can inhibit ER stress in ox-LDL-treated endothelial cells.

### 3.3. Melatonin Sustains Mitochondrial Function in ox-LDL-Treated Endothelial Cells

**Figure 3:**
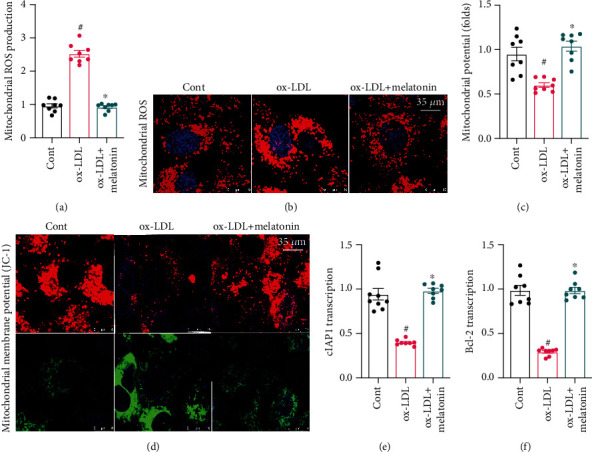
Melatonin sustains mitochondrial function in ox-LDL-treated endothelial cells. (a, b) Mitochondrial ROS production was examined using a mitochondrial ROS probe. HUVECs were treated with melatonin in the presence of ox-LDL. (c, d) Mitochondrial membrane potential was determined using the JC-1 probe. (e, f) qPCR was used to measure cIAP1 and Bcl-2 transcription in response to ox-LDL treatment with or without melatonin. ^#^*p* < 0.05 vs. the control group, ^∗^*p* < 0.05 vs. the ox-LDL group.

In addition to ER stress, we also examined the effects of ox-LDL on mitochondrial dysfunction. ox-LDL significantly increased mitochondrial oxidative stress as indicated by a mitochondrial ROS probe assay (Figures 3(a) and 3(b)). Interestingly, melatonin treatment strongly inhibited mitochondrial ROS production in endothelial cells. Next, we examined the regulatory effects of melatonin on mitochondrial membrane potential. As shown in Figures 3(c) and 3(d), compared to the control group, ox-LDL treatment disrupted mitochondrial membrane potential, as indicated by decreased red JC-1 probe fluorescence. Interestingly, melatonin treatment significantly reversed this disruption of mitochondrial membrane potential. ox-LDL treatment also significantly decreased the expression of mitochondrial protective factors Bcl-2 and c-IAP1 (Figures 3(e) and 3(f)), and melatonin treatment again reversed this decrease. Taken together, these results indicate that melatonin protected mitochondrial function in endothelial cells.

### 3.4. Melatonin Inhibits Oxidative Stress in ox-LDL-Treated Endothelial Cells

**Figure 4:**
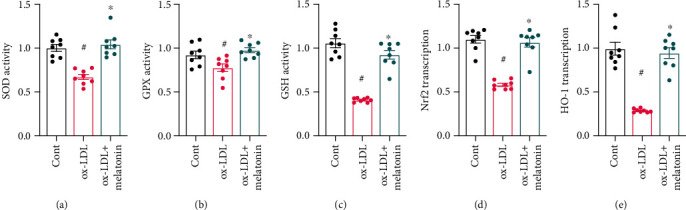
Melatonin inhibits oxidative stress in ox-LDL-treated endothelial cells. (a–c) ELSIAs were used to measure the activity of antioxidative enzymes such as GSH, GPX, and SOD. (d, e) qPCR was used to analyze Nrf2 and HO-1 transcription in response to ox-LDL treatment with or without melatonin. ^#^*p* < 0.05 vs. the control group, ^∗^*p* < 0.05 vs. the ox-LDL group.

In addition to ER stress and mitochondrial dysfunction, we also explored whether ox-LDL induces oxidative stress that contributes to endothelial dysfunction. As shown in Figures 4(a)–4(c), compared to the control group, ox-LDL significantly inhibited the activity of antioxidative enzymes. Interestingly, melatonin treatment reversed this inhibition of antioxidative enzymes such as GSH, SOD, and GPX. Upstream regulatory mechanisms underlying melatonin-induced antioxidative effects were then examined further. Nrf2 and HO-1 have been identified as the primary antioxidative signaling molecules in endothelial cells. qPCR indicated that ox-LDL treatment significantly decreased Nrf2 and HO-1 transcription (Figures 4(d) and 4(e)), while melatonin treatment restored Nrf2 and HO-1 levels. Taken together, these results demonstrate that melatonin could reduce oxidative stress in ox-LDL-treated endothelial cells.

### 3.5. Melatonin Inhibits the JNK/Mff Signaling Pathway in ox-LDL-Treated Endothelial Cells

**Figure 5:**
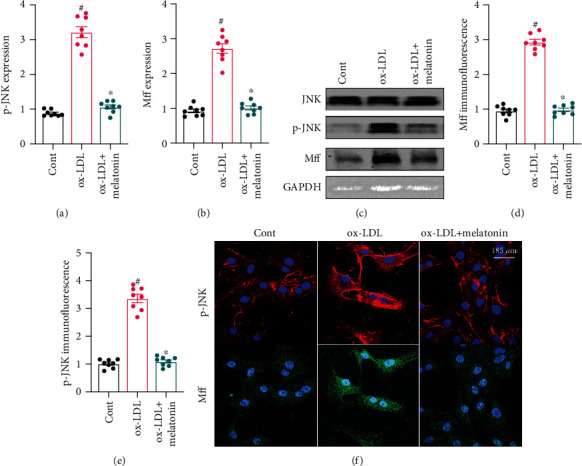
Melatonin inhibits the JNK/Mff signaling pathway in ox-LDL-treated endothelial cells. (a–c) Proteins were isolated from treated HUVECs and p-JNK and Mff levels were evaluated using western blots. (d, f) Immunofluorescence was used to measure o-JNK and Mff expression in HUVECs treated with melatonin in the presence of ox-LDL. ^#^*p* < 0.05 vs. the control group, ^∗^*p* < 0.05 vs. the ox-LDL group.

Finally, we evaluated an upstream signaling pathway that might underlie ox-LDL-induced endothelial dysfunction. Recent studies indicate that the JNK/Mff signaling pathway is associated with inflammation response, cell apoptosis, and oxidative stress in endothelial cells in the context of cardiac ischemia-reperfusion injury. Based on this finding, we examined whether melatonin sustained endothelial function by inhibiting the JNK/Mff signaling pathway. As shown in Figures 5(a)–5(c), western blots demonstrated that ox-LDL activated the JNK pathway as evidenced by increased JNK phosphorylation in endothelial cells. Mff expression was also upregulated in ox-LDL-treated endothelial cells (Figures 5(a)–5(c)). These results indicate that ox-LDL treatment activates the JNK/Mff signaling pathway in endothelial cells. In addition, melatonin inhibited JNK phosphorylation and Mff upregulation in ox-LDL-treated endothelial cells. Immunofluorescence confirmed this finding. As shown in Figures 5(d)–5(f), compared to the control group, ox-LDL increased JNK and Mff immunofluorescence intensity, and melatonin returned both markers to near-normal levels. Overall, these data indicate that melatonin can inhibit the JNK/Mff signaling pathway in ox-LDL-treated endothelial cells.

## 4. Discussion

In this study, we found that ox-LDL caused endothelial dysfunction characterized by decreases in cell viability, impaired proliferative capacity, blunted migratory ability, and increases in cell apoptosis rate. Endothelial dysfunction was associated with ER stress, mitochondrial damage, and oxidative stress. At the molecular level, expression of CHOP, PERK, and GRP78 was increased in ox-LDL-treated endothelial cells. Moreover, decreased mitochondrial membrane potential, increased mitochondrial ROS levels, and downregulation of mitochondrial protective factors were observed in ox-LDL-treated endothelial cells. In addition, ox-LDL reduced the activity of antioxidative enzymes and prevented activation of antioxidative signals. Interestingly, melatonin treatment attenuated ox-LDL-induced ER stress, as evidenced by decreased expression of CHOP, PERK, and GRP78. Mitochondrial function and oxidative stress also improved after melatonin administration in ox-LDL-treated endothelial cells. Finally, we found that melatonin protected endothelial cells against ER stress and mitochondrial dysfunction after ox-LDL treatment by inhibiting the JNK/Mff signaling pathway. Taken together, our results demonstrate that ox-LDL promoted endothelial dysfunction by activating ER stress, mitochondrial damage, oxidative stress, and the JNK/Mff signaling pathway. Furthermore, melatonin effectively sustained endothelial cell function by improving ER homeostasis, mitochondrial performance, redox balance, and JNK/Mff axis activity.

ER stress is an adaptive response that affects the quality and quantity of proteins within endothelial cells [57]. When the ER detects the presence of unfolded proteins, CHOP, PERK, and GRP78 expressions increase [58]. Those three proteins then migrate from the ER to the nucleus to interrupt protein transcription more broadly [59]. However, excessive ER stress promotes activation of stress-related proteins such as caspase-12, which is involved in the initiation of cell apoptosis [60]. The relationship between ER stress and endothelial dysfunction has been described in detail in previous studies. For example, ER stress induces interleukin-6 (IL-6) release from endothelial cells, which damages the endothelial barrier [61]. Hypoxia-mediated endothelial cell apoptosis also seems to be associated with ER stress [62]. Additionally, laminar flow-mediated endothelial protection is associated with inhibition of ER stress through the PI3K/Akt signaling pathway [63]. Our present results further indicate that ER stress contributes to endothelial dysfunction when ox-LDL levels are elevated.

In addition to ER stress, mitochondrial damage or stress also may play a role in endothelial dysfunction. Mitochondria produce ATP to support endothelial cell growth and metabolism [64, 65]. Decreased mitochondrial function has been identified as an early event during endothelial dysfunction [66]. In addition, ATP produced by the mitochondria plays a key regulatory role in endothelia-dependent angiogenesis [67]. In the present study, we found that ox-LDL-induced mitochondrial damage was characterized by mitochondrial membrane potential disruption, mitochondrial ROS overproduction, and downregulation of mitochondrial protective factors. These findings are in accordance with the previous studies [68].

Our data also demonstrate that ER stress and mitochondrial damage could be attenuated by melatonin in endothelial cells. At the molecular level, the JNK/Mff signaling pathway played a role in melatonin's ability to sustain ER homeostasis and mitochondrial function after ox-LDL treatment. Zhou et al. [27] were the first to report a role for the JNK/Mff signaling pathway in the context of cardiac ischemia-reperfusion injury. In that study, reperfusion injury induced JNK phosphorylation and thus promoted Mff transcription, which was followed by mitochondrial fragmentation, intracellular oxidative stress, and endothelial cell apoptosis [27]. In the present study, we found that the JNK/Mff axis was also activated by ox-LDL and might contribute to ox-LDL-induced endothelial dysfunction. In addition, melatonin treatment inhibited JNK/Mff signaling pathway activity. This finding provides novel insight into the pathological mechanisms underlying ox-LDL-related endothelial dysfunction as well as potential treatments.

In summary, we found that ox-LDL triggered endothelial dysfunction by inducing ER stress, mitochondrial damage, and oxidative stress. Furthermore, melatonin treatment was able to sustain endothelial viability by inhibiting the JNK/Mff signaling pathway and attenuating damage to the ER and mitochondria. Our study thus identified potential targets for clinical treatments that might protect endothelial function.

## Data Availability

All data generated or analyzed during this study are included in the published article.
